# Medicines Received

**Published:** 1866-12

**Authors:** 


### Editorial Department.

### Medicines Received.

We have to acknowledge the reception of a box of medicines from Reed, Carnrick & Andrus, comprising the following preparations:—Elixir cinchona, iron and strychnia; elixir bark and protoxide of iron; elixir calisaya, iron and bismuth; elixir bark and pyrophosphate of iron; elixir pyrophosphate of iron and soda; elixir valerianate of ammonia; elixir valerianate of ammonia and quinine; elixir valerianate of strychnia; effervescing magnesian aperient; citrate of bismuth; tannate of bismuth; solution gutta percha. We have not tested their curative virtues, but they are elegant and palatable preparations, and contain the properties of the several drugs scientifically combined, in proportions suitable for physician's prescriptions. These remedies are becoming quite fashionable, and the demand for them by the public is so positive that it is not always easy for the physician to make choice of the former modes of administering similar medicines. If they are less efficient, they are so much more agreeable that their employment is becoming extensive.

Reed, Carnrick & Andrus, and Howell & Onderdonk have fully supplied the market in Buffalo, and most of our druggists can furnish their preparations. We do not propose to advise physicians to discontinue the former modes of preparing and prescribing remedies; it is probable that all these medicines can bs used in less expensive forms, and prove as efficacious in the cure of disease; at the same time it cannot be doubted that the above preparations possess some advantages over administering these drugs in their more crude forms. It appears with some of them, that elegance and palatableness are their chief virtues, but most which have been tested show that they are efficient and valuable in the cure of disease, as valuable as the same drug in other and less agreeable forms. The effort to make our remedies less unpleasant, has been crowned with success, and physicians are under many obligations to the manufacturing chemists for furnishing remedies in agreeable and efficient forms.

